# Supraphysiological Role of Melatonin Over Vascular Dysfunction of Pregnancy, a New Therapeutic Agent?

**DOI:** 10.3389/fphys.2021.767684

**Published:** 2021-11-16

**Authors:** Francisco J. Valenzuela-Melgarejo, Constanza Lagunas, Fabiola Carmona-Pastén, Kevins Jara-Medina, Gustavo Delgado

**Affiliations:** Laboratory of Molecular Cell Biology, Department of Basic Sciences, Universidad del Bío-Bío, Campus Fernando May, Chillán, Chile

**Keywords:** melatonin, hypertension, pregnancy, vascular, preeclampsia

## Abstract

Hypertension can be induced by the disruption of factors in blood pressure regulation. This includes several systems such as Neurohumoral, Renin-angiotensin-aldosterone, the Circadian clock, and melatonin production, which can induce elevation and non-dipping blood pressure. Melatonin has a supraphysiological role as a chronobiotic agent and modulates vascular system processes via pro/antiangiogenic factors, inflammation, the immune system, and oxidative stress regulation. An elevation of melatonin production is observed during pregnancy, modulating the placenta and fetus’s physiological functions. Their impairment production can induce temporal desynchronization of cell proliferation, differentiation, or invasion from trophoblast cells results in vascular insufficiencies, elevating the risk of poor fetal/placental development. Several genes are associated with vascular disease and hypertension during pregnancy via impaired inflammatory response, hypoxia, and oxidative stress, such as cytokines/chemokines IL-1β, IL-6, IL-8, and impairment expression in endothelial cells/VSMCs of HIF1α and eNOS genes. Pathological placentas showed differentially expressed genes (DEG), including vascular genes as CITED2, VEGF, PL-II, PIGF, sFLT-1, and sENG, oncogene JUNB, scaffolding protein CUL7, GPER1, and the pathways of SIRT/AMPK and MAPK/ERK. Additionally, we observed modification of subunits of NADPH oxidase and extracellular matrix elements, i.e., Glypican and Heparanase and KCa channel. Mothers with a low level of melatonin showed low production of proangiogenic factor VEGF, increasing the risk of preeclampsia, premature birth, and abortion. In contrast, melatonin supplementation can reduce systolic pressure, prevent oxidative stress, induce the activation of the antioxidants system, and lessen proteinuria and serum level of sFlt-1. Moreover, melatonin can repair the endothelial damage from preeclampsia at the placenta level, increasing PIGF, Nrf-2, HO-1 production and reducing critical markers of vascular injury during the pregnancy. Melatonin also restores the umbilical and uterine blood flow after oxidative stress and inhibits vascular inflammation and VCAM-1, Activin-A, and sEng production. The beneficial effects of melatonin over pathological pregnancies can be partially observed in normal pregnancies, suggesting the dual role of/over placental physiology could contribute to protection and have therapeutic applications in vascular pathologies of pregnancies in the future.

## Introduction

The control of blood pressure results from the contribution of several tissues and neural circuits via the multifactorial interaction of several physiological factors such as the heart rate, cardiac output, and peripheral resistance. The peripheral resistance determines the peripheral blood circulation, dependent on the arterial and venous tone. The chronic elevation of blood pressure (persistently raised pressure, >140 mmHg/90 mmHg) or hypertension is a severe medical condition associated with elevated risk factors for morbidity and mortality worldwide, a major cardiovascular risk factor. These pathologies are present between 20 and 25% of the population, or about 1.13 billion people worldwide (World Health Organization, and World Health Organization, 2013), and affect about 8% of reproductive-aged women, representing about 688 million women (Mupfasoni et al., 2018; Braunthal and Brateanu, 2019). The severe expression of hypertension is named malignant-hypertension or accelerated-hypertension, affecting about 2-7 cases per 100,000 habitants. This rate increases every year (Shantsila and Lip, 2017), suggesting that this pathology and its more extreme variations have become a massive health problem in terms of morbidity and mortality worldwide.

**FIGURE 1:**
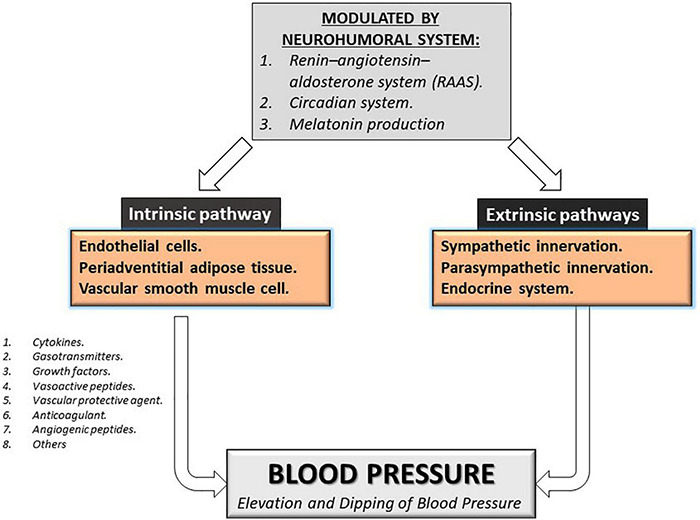
Modulation of blood pressure. Intrinsic and extrinsic pathways can modulate blood pressure. The principal regulator of both pathways is the neurohumoral system Renin-Angiotensin-aldosterone system, the circadian system, and melatonin production.

Several factors modulate vascular circulation and blood pressure, which can be divided into intrinsic and extrinsic pathways, modulating the vascular tone, coagulation, and the vascular system’s flow. Intrinsic regulation pathways involve the paracrine production of endothelial cells, periadventitial adipose tissue, and vascular smooth muscle cell. The extrinsic regulation factor involves neuronal regulation such as sympathetic/parasympathetic innervation and the humoral secretion from the endocrine system. The intrinsic occurs via the paracrine liberation of cytokines, gasotransmitters, growth factors, vasoactive peptides, vascular protective agents, anticoagulant, angiogenic peptides, and others, which maintain the vasomotor and mitogenic balance required for an adequate vascular tone in the peripheral circulation (Konukoglu and Uzun, 2017; Gheibi et al., 2018; Oparil et al., 2018). These complex interactions require a supraphysiological regulation that includes the participation of the neurohumoral system that includes the renin-angiotensin-aldosterone system (RAAS), the circadian system, and melatonin production by the pineal gland see Figure 1 (Baker and Kimpinski, 2018; Nakashima et al., 2018; Oparil et al., 2018; Zuo and Jiang, 2020). Disruption of the intrinsic or extrinsic factors involved in blood pressure regulation can induce elevation and non-dipping blood pressure resulting in damage over vascular cells or tissues. Moreover, these factors can be affected by nutrition, environment, fetal programming, adiposity, diet, sodium and potassium intake, alcohol intake, smoking, physical activity, air pollution, and stress which give multivariable causes and expressions for hypertension (NCD Risk Factor Collaboration (Ncd-RisC), 2017). However, multifactorial gene-environment etiology is associated with 90–95% of patients with primary hypertension, besides showing an association with a genetic component in about 35-50% of patients, suggesting the relevance of finding new pathways and new molecular markers to help predict the risk of morbidity and mortality by hypertension (Oparil et al., 2018).

Melatonin has a role as a chronobiotic agent synchronizing the circadian system and plays a supraphysiological role in modulating other vascular system processes via modulation of inflammation, the immune system, and oxidative stress. This role was first described in the late-1960s in Pinealectomy in rats (Zanoboni and Zanoboni-Muciaccia, 1967). This study observed an increase of 30% in blood pressure (hypertension) at 15 days after surgery (Zanoboni and Zanoboni-Muciaccia, 1967). Melatonin supplementation can partially revert the harmful effects of this hypertension, as lipoperoxidation, hydroxyl radical generation, superoxide anion radical, and inducing antioxidant capacity via increased glutathione (GSH) content (Mukherjee et al., 2010). Moreover, melatonin plays a protective role in restoring hemodynamic parameters after myocardial injury, explaining blood pressure reduction in pathological conditions (Mukherjee et al., 2010), induced arterial vasorelaxation after vasoconstrictor treatment, and prevented the vasoconstriction at the level of cerebral arteries (Torres-Farfan et al., 2008; Qiu et al., 2018). Patients treated with melatonin supplementation reduce by about 10% in the MAP and SBP, not altering the heart rate (Bazyar et al., 2021), suggesting a protective role over the cardiovascular function of melatonin hormone by their capacity antioxidant and hypotensive role over the cardiovascular system.

The cardiovascular disease of the mother/offspring induces an adverse intrauterine environment which gives outputs such as fetal hypoxia, intrauterine growth restriction, gestational hypertension, and Preeclampsia. During normal pregnancy, melatonin production increase depending on gestational age and falls immediately after delivery (Nakamura et al., 2001; Ejaz et al., 2020). The impaired melatonin production is associated with complications during pregnancy, such as severe Preeclampsia, hypertension, and proteinuria. During severe Preeclampsia, Melatonin levels in women are reduced (Dou et al., 2019). However, melatonin supplementation can reduce oxidative stress and hypertension during pregnancy. Suggesting that melatonin production during pregnancy maintains cardiovascular health, reducing premature birth and abortion (Valenzuela et al., 2015; de Chuffa et al., 2019).

However, potentially, another genetic component can work during hypertension, and these genes can be modulated by melatonin. For this purpose, we searched the Differentially Expressed Genes (DEG) during hypertension detected in the tone regulation by vascular smooth muscle contraction. We found 36 upregulated and downregulated genes in vascular smooth muscle contraction pathways during hypertension see Table 1. This suggests that there are a number of pathways involved in this pathology and the complex pathways involved in vascular smooth muscle.

**TABLE 1:** Genes into vascular smooth muscle altered by hypertension that could alter contraction pathways.

Differentially expressed genes associated with Hypertension *(N* = *36 genes)*			
*ARAF*	*GUCY1A3*	*NPR2*	*PRKCG*
*GNAS*	*GUCY1B3*	*PLA2G2A*	*PPP1CC*
*RAF1*	*ITPR1*	*PLA2G4A*	*RHOA*
*ARHGEF11*	*ITPR2*	*PLA2G6*	*RAMP1*
*ADCY2*	*ITPR3*	*PLA2G2C*	*RAMP2*
*ADCY5*	*MAPK1*	*PLA2G5*	*RAMP3*
*ADCY6*	*MAP2K1*	*PLCB4*	
*ADRA1D*	*MAP2K2*	*KCNMB4*	
*AGTR1A*	*MYLK2*	*PRKCD*	
*CALM3*	*NPR1*	*PRKCH*	

*Analyses of datasets from the GEO database (http://www.ncbi.nlm.Nih.gov/geo) available for hypertension were performed in the GEO database (n = 73). We excluded the platforms without gene accession numbers, incomplete incoming information, and results of peripheral blood. Platforms GSE74288, GSE113613, GSE89073, GSE105114, GSE105114, GSE105114, GSE84704, GSE53363, GSE72707, GSE72181, GSE64613, GSE69601, GSE46863, GSE53408, GSE45927, GSE59437-BRAIN, GSE50833, GSE48936, GSE43292, GSE40182, GSE26671, GSE30428, GSE24988, GSE19817, GSE16624, and GSE5488 were visualized using the GEO Profile graphics a web tool to compare the two groups using Benjamin and Hochberg false discovery rate methodology, using the parameter by default (logFC ≥ 1 and adj. P < 0.05). For the functional analysis, we used the Kyoto Encyclopedia and Genes and Genomes (KEGG) and selected vascular smooth muscle contraction pathways. The gene expression profile was combined and identified with Venn Diagram, we then undertook the identification of DEGs.*

## Hypertension and Pregnancy

The American College of Obstetricians and Gynecologists (ACOG) defines hypertension during pregnancy as a pressure ≥ 140/90 mm Hg for systolic and/or diastolic BP (Bello et al., 2021). Following this criterion, hypertension can affect about 8% of women of reproductive age and present in about 10% of pregnancies (Braunthal and Brateanu, 2019). During the pregnancy, about 4,3% corresponded to chronic hypertension, and 6% were defined as gestational hypertension, which during the pregnancy elevates the negative outputs for the mother and fetus, such as preeclampsia, preterm birth, and the baby being small for gestational age (Bello et al., 2021). Impaired placentation is the principal cause of complications in pregnancy. It causes several negative outputs, including hypertension, and their severe expression occurs in about 2% and induces about 16% of all maternal deaths suggesting the relevance of prevention of hypertension and preeclampsia (Romero et al., 2011; Poniedziałek-Czajkowska et al., 2021).

Preeclampsia involves the vasculature due to the impaired transformation of the spiral arteries and the reduced perfusion to the fetus and the placenta, with an elevation of oxidative and endoplasmic reticulum stress and finally, inducing a fetal growth restriction. The systemic stress shoddy remodeling of the uteroplacental spiral arteries release placental factors to maternal circulation, increasing the maternal inflammatory response and oxidative stress such as high production of proinflammatory cytokines/chemokines such as IL-1β, IL-6, and IL-8 (Nunes et al., 2019; Valencia-Ortega et al., 2019; Poniedziałek-Czajkowska et al., 2021; Spence et al., 2021). Moreover, the impaired invasion of the uterine wall by trophoblast induce hypoxia, and produce a modification of placental secretion of critical angiogenic and antiangiogenic factors such as vascular endothelial growth factor (VEGF) (Frigato et al., 2009) and Placental lactogen (PL-II) member of the prolactin gene family. These vascular factors and their impaired secretion have been proposed to predict risk during pregnancy (Wang and Zhao, 2010; Valenzuela et al., 2012; Lenke et al., 2019). Besides, VEGF and placental growth factor (PlGF) induce placental angiogenesis via activation of VEGFR-1/Flt-1 and VEGFR-2/KDR, and both factors increase endothelial cell adhesion, chemotaxis and increase angiogenesis (Helske et al., 2001; Poniedziałek-Czajkowska et al., 2021).

Several antiangiogenic factors are highly secreted during pathological pregnancies, such as Fms-like tyrosine kinase-1 (sFlt-1), which is the soluble secretion of VEGF Receptor-1, and their secretion by villous cytotrophoblasts cells induced by hypoxia during Preeclampsia via activation of HIF1α. Another antiangiogenic factor secreted during Preeclampsia is soluble Endoglin (sEng), which reduces the proangiogenic and vasodilator effects of Endoglin. Finally, the elevated production of sFlt-1 and sEng and the decrease of VEGF and PIGF secretion induce an impairment of endothelial function, causing preeclampsia (De Oliveira et al., 2013; Shah and Khalil, 2015; Poniedziałek-Czajkowska et al., 2021).

The impaired production of pro/antiangiogenic factors, added to ROS stress, low activity of endothelial nitric oxide synthase (eNOS), low production of nitric oxide (NO) is characteristic of preeclampsia. This last gasotransmitter is a potent vasodilator, which is critical for appropriate trophoblast remodeling of spiral arteries (Poniedziałek-Czajkowska et al., 2021).

Sirtuin 1 (SIRT1) plays a critical role during pregnancy, via a stress-response and chromatin-silencing factor associated with a NAD-dependent histone deacetylase activity associated with DNA replication, DNA repair, an extension of life span, and reduction of apoptosis. Moreover, SIRT1 reduces the release of proinflammatory cytokines via inhibition of NF-kappa-B signaling (Chen et al., 2005; Abdelmohsen et al., 2007; Poniedziałek-Czajkowska et al., 2021). This signaling pathway is induced by AMP-activated protein kinase or AMPK (Yi et al., 2021). The SIRT1 expression in the placenta and the plasmatic concentration showed a low level in preeclamptic women at 20-25 weeks of gestation (Yin et al., 2017; Viana-Mattioli et al., 2020; Poniedziałek-Czajkowska et al., 2021).

The AMPK pathway is activated by increasing AMP levels and decreasing ATP levels (low energy). The AMPK is a heterotrimeric protein composed of αβγ subunits and expressed ubiquitously. The catalytic α subunit has two isoforms, but the α1 subunit (AMPK1) is the predominant subunit in Vascular smooth muscle cells (VSMCs) and endothelial cells, playing a role in vascular remodeling in atherosclerosis and pulmonary hypertension (Zhao et al., 2021). The AMPK1 gene is expressed in uterine arteries and placenta, and their expression increased during pregnancy exposed to hypoxia or models of preeclampsia (Skeffington et al., 2016), which is associated with the early onset of preeclampsia in humans (Liu et al., 2020). The pharmacological activation of AMPK1 can increase capacity to reduce the fetal restriction induced by hypoxia by an increase of uterine artery flow by approximately twofold (Lane et al., 2020), and these increases of blow flow can be stimulated by dependent via of NO (≈40%) and independent pathways (Skeffington et al., 2016), suggesting this protein plays a critical role in the vascular system of the placenta.

Another genetic component could potentially work during vascular pathologies such as preeclampsia. For example, an analysis of the transcript levels of 14,040 genes in the placenta from mothers with normotensive symptoms or hypertension during pregnancy was undertaken by Cox et al. (2019). The present re-analysis of data available in the GEO database^1^ aims to find genes dysregulated at the vascular level that are associated with preeclampsia. Several pathways identified in the Kyoto Encyclopedia of Genes and Genomes (KEGG) can modulate vascular function. We identified the genes that were the most enriched and associated with the KEGG pathway as “negative regulation of vascular smooth muscle cell proliferation,” “vasculature development,” “vascular endothelial growth factor receptor signaling pathways,” “vascular wound healing,” “coronary vasculature development,” “vasculogenesis,” and “vascular endothelial growth factor receptor signaling pathways” see Table 2. Among the genes we identified, CITED2 plays a role in vasculogenesis, a critical gene in the cellular response to hypoxia, and showed the capacity to inhibit HIF1α activation and cellular response to hypoxia (Berlow et al., 2017). During preeclampsia, the activation of Endoplasmatic Reticulum stress induces the secretion of extracellular vesicles and the inhibition of CITED2 expression in the placenta (Collett et al., 2018). The CBP/p300-interacting transactivator, with glu/asp-rich c-terminal domain-2 or CITED2, plays a role in trophoblast differentiation and is expressed in vascular endothelial trophoblast cells. Their deletion induces placental malformation, decreasing placenta and embryo weight and reducing the number of placental malformation syncytiotrophoblasts, resulting in embryo death (Moreau et al., 2014; Imakawa et al., 2016). This suggests that these genes may be a new target of study. Similarly, another transcriptional via detected in the vasculogenesis pathway is oncogene JUNB (a subunit of AP1 factor), and their impaired expression in the placenta can elevate the risk of Preeclampsia. For example, the elevation of JUNB expression during pregnancy gives a high expression of Phosphatase and tensin homolog or PTEN protein and a reduction of approximately 50% of trophoblast invasiveness (Xue et al., 2020). Similarly, other studies in the cell line of the trophoblast suggest that elevated expression of JUNB can elevate the proliferation, migration, and stimulation of angiogenesis (Zou et al., 2018). In contrast, JUNB is downregulated in placenta-derived Mesenchymal Stromal Cells from the woman with preeclampsia (Nuzzo et al., 2017), suggesting this gene plays a critical role during pregnancy and preeclampsia.

**TABLE 2:** Functional annotation pathways modified by hypertension in placenta.

Negative Regulation of Vascular Smooth Muscle Cell Proliferation	Vasculature Development	Vascular Endothelial Growth Factor Receptor Signaling Pathway		Vascular Wound Healing	Coronary Vasculature Development	Vasculogenesis
**GPER1** *G protein-coupled estrogen receptor 1*	**B9D1** *B9 domain containing 1*	**CRK** *CRK proto-oncogene, adaptor protein*	**NCKAP1L** *NCK associated protein 1 like*	**CD34** *CD34 molecule*	**SMAD6** *SMAD family member 6*	**CITED2** *Cbp/p300 interacting transactivator with Glu/Asp rich carboxy-terminal domain 2*
**CNN1** *calponin 1*	**RIC8A** *RIC8 guanine nucleotide exchange factor A*	**FYN** *FYN proto-oncogene, Src family tyrosine kinase*	**SRC** *SRC proto-oncogene, non-receptor tyrosine kinase*	**ADIPOR2** *Adiponectin receptor 2*	**DCTN5** *dynactin subunit 5*	**JUNB** *JunB proto-oncogene, AP-1 transcription factor subunit*
**CDKN1B** *cyclin dependent kinase inhibitor 1B*	**ANP32B** *acidic nuclear phosphoprotein 32 family member B*	**ROCK2** *Rho associated coiled-coil containing protein kinase 2*	**WASF2** *WAS protein family member 2*	**HPSE** *heparanase*	**DNM2** *dynamin 2*	**SOX17** *SRY-box 17*
**TGFB3** *transforming growth factor beta 3*	**CALCA** *calcitonin related polypeptide alpha*	**ACTG1** *actin gamma 1*	**CYFIP1** *cytoplasmic FMR1 interacting protein 1*	**NDNF** *neuron derived neurotrophic factor*	**GPC3** *glypican 3*	**CUL7** *cullin 7*
	**ZFAND5** *zinc finger AN1-type containing 5*	**BMPR2** *bone morphogenetic protein receptor type 2*	**MAPK14** *mitogen-activated protein kinase 14*		**MEGF8** *multiple EGF like domains 8*	**GJC1** *gap junction protein gamma 1*
		**CYBA** *cytochrome b-245 alpha chain*	**MAPKAPK2** *mitogen-activated protein kinase-activated protein kinase 2*		**MYH10** *myosin heavy chain 10*	**GLMN** *glomulin, FKBP associated protein*
		**ELMO1** engulfment and cell motility 1	***NCF4*** *neutrophil cytosolic factor 4*		**PLXND1** *plexin D1*	**HEG1** *heart development protein with EGF like domains 1*
		**FOXC1** *forkhead box C1*	**VEGFA** *vascular endothelial growth factor A*		**PRICKLE1** *prickle planar cell polarity protein 1*	**VEGFA** *vascular endothelial growth factor A*
		**HSPB1** *heat shock protein family B (small) member 1*	***VAV1*** *vav guanine nucleotide exchange factor 1*			
		**ITGAV** *integrin subunit alpha V*	**NCF1** *neutrophil cytosolic factor 1*			
		**ITGB3** *integrin subunit beta 3*	**PIK3CB** *phosphatidylinositol-4,5-bisphosphate 3-kinase catalytic subunit beta*			
		**MAPK13** *mitogen-activated protein kinase 13*	**PIK3R1** *phosphoinositide-3-kinase regulatory subunit 1*			
		**RAC1** *ras-related C3 botulinum toxin substrate 1 (rho family, small GTP binding protein Rac1)*	***PTK2B*** *protein tyrosine kinase 2 beta*			
		**SULF1** *sulfatase 1*				

*The analysis of the transcript levels of 14,040 genes in the placenta from mothers with normal or hypertension during pregnancy, obtained by Cox et al. (2019). Re-analysis of data available in GEO (https://www.ncbi.nlm.nih.gov/gds) to find genes dysregulated at the vascular level, and perform functional analysis of differentially expressed genes characterized by gene ontology (GO) and pathway enrichment analyses (DAVID Bioinformatics Resources 6.8, NIAID/NIH). Our approximation gives co-expression network analysis by functional pathways that are modified in the placenta by hypertension and have a role in vascular health. The most enriched KEGG pathway saw more negative regulation of vascular smooth muscle cell proliferation, vasculature development, vascular endothelial growth factor receptor signaling pathways, vascular wound healing, coronary vasculature development, vasculogenesis, and vascular endothelial growth factor receptor signaling pathways.*

A differentially expressed gene detected in Table 2 is Cullin 7, or CUL7, a scaffolding protein expressed in all tissues and associated with ubiquitin ligase. Hypoxia during pathological pregnancies reduces CUL7 expression in the villous trophoblast and syncytiotrophoblast, inducing impaired placental development (Tsunematsu et al., 2006; Fahlbusch et al., 2012).

Another dysregulated gene detected in Table 2 is the Vascular endothelial growth factor type A (VEGFA), which is critical during pregnancy for endothelial cell proliferation, migration, and angiogenesis. That is elevated in maternal serum in PE cases at 30 weeks and is sequestered during preeclampsia by excessive production of sFLT-1, resulting in endothelial dysfunction, suggesting an excess of VEGF production might play a role in preeclampsia by VEGF toxicity and stimulation of sFLT-1 production (Jena et al., 2020). Women with preeclampsia showed an elevated level of SRC protein, but the activation by the phosphorylation in the Tyr-416 residue was lowered, suggesting a low activation compared with normal women. Downstream genes activated by SRC, including ERK1/2, p38, and JNK showed low phosphorylation in preeclampsia, demonstrating the inactivation of SRC or c-SRC, which are critical for trophoblast development and differentiation. MAPK14 or p38-alpha is critical for trophoblast development and differentiation, and their activation stimulates the PPARγ pathway. This pathway induces the vascularization and expression of Syncytin-1, a critical element in placentation. In contrast, the human placenta from Preeclampsia showed a low level of expression of MAPK14- PPARγ- Syncytin-1 genes (Ruebner et al., 2012). Fyn is an oncogene that plays a role in the ERK via transduction, stimulating the expression of the KCa3.1 channel, an endothelial vasodilator, and inhibiting blood pressure increases during the pregnancy. However, another study observed the downregulation of this pathway during preeclampsia (Choi et al., 2019).

Another gene observed in Table 2 is the Neutrophil cytosolic factor 4 or Ncf4, a subunit of NADPH oxidase, which is expressed in trophoblast cells after implantation (Gomes et al., 2012). This activity is the primary source of placental oxidative stress, which is a characteristic of preeclampsia. Similarly, another member detected is the subunit CYBA gene elevated in preeclampsia (Gomes et al., 2012; Trifonova et al., 2014), suggesting that the NADPH oxidase activity plays a critical role during the oxidative stress observed in the pregnancy. Moreover, elevated NADPH oxidase activity can modulate the production of sFlt-1 and PlGF, suggesting the critical role of this activity in developing preeclampsia (Hernandez et al., 2021).

The human placenta produces proteoglycans and the Glypican family are a member of these macromolecules. They are present in the Plasmatic Membrane by GPI anchor and can interact with VEGF. Glypican-3 (GPC3) modified their expression in pathological placentas (Table 2), playing an essential role in proliferation and differentiation and expressed in the human placenta. Low content is detected at the third trimester of gestation in samples of placenta and maternal serum from pregnancies diagnosed with Fetal Growth Restriction and preeclampsia (Chui et al., 2012; Gunatillake et al., 2019; Shimizu et al., 2019). Myosin heavy chain-10 or MYH10 modulates cell migration and invasion with preimplantation factor peptide or PIF to promote the implantation and remodeling of the uterine wall (Yang et al., 2018).

Another member of the Plasmatic Membrane observed in Table 2 is the Plexin D1 or PLXND1, which plays a role in signaling endothelial cells and their impaired expression is associated with vascular disease, inducing atherosclerotic lesions by macrophages and inhibiting angiogenesis via stimulation of soluble Flt-1, an inhibitor of VEGF (Zhang et al., 2021). Similarly, we detected a differential expression in the pathological placenta of SMAD family member-6 or SMAD6 (see Table 2). When expressed postnatally this gene can modulate endothelial gene expression and participates in vascular development. Their mutation is associated with hypertension in children associated with renal arterial occlusive disease (Viering et al., 2020).

Preeclampsia is a two-stage disease with abnormal placentation and placental hypoxia by the impaired remodeling of the uterine wall. This affects the maternal endothelium and the production of Endothelial progenitor cells (EPC). The EPC circulating in maternal blood is characterized by CD34 antigen expression, and is used as a preeclampsia marker, related to vascular wound healing, and detected in pathological placenta (Table 2). Over 20 weeks gestation, women with preeclampsia showed a high level of Endothelial progenitor cells (CD34+) than the normotensive women (Brown et al., 2019). In contrast, in early pregnancy a low level of CD34 + cells have been observed (Laganà et al., 2017), suggesting that they are a marker dependent on gestational week and pathology during pregnancy.

Adiponectin receptor 2 (ADIPOR2) is a G protein-coupled receptor expressed in the embryo and placenta. ADIPOR2 induces a low proliferation via inactivation of the JNK pathway in the placenta and stimulates the lipid metabolism in the embryo. The human placenta is an independent source of Adiponectin, and their production in the placenta has proinflammatory effects and antiproliferative effects during the first trimester over trophoblast cells. However, several reports suggest a contradictory level of Adiponectin can be potentially produced by an adiponectin resistance state (Barbe et al., 2019).

Heparanase (HPSE) is a dysregulated gene observed in the placenta (Table 2) that plays a role in vascular wound healing, cleaving the heparan chain on the cell surface. Their products bind to sFLT-1, which promotes proliferation and invasion of trophoblast cells during early pregnancy (Che et al., 2018). However, their expression is elevated by hypoxia during preeclampsia, and this elevated activity stimulates the hypoxia-induced sFLT-1 release and inhibition of the proangiogenic function of VEGF (Ginath et al., 2015; Eddy et al., 2019). The bone morphogenetic protein receptor type-II or BMPR2 is a plasmatic membrane protein that transduces extracellular signals through the formation of heteromeric complexes, and their dysregulation plays a role during pulmonary hypertension vascular remodeling and endothelial dysfunction (Machado et al., 2003; D’Amico et al., 2018). We detected the dysregulated expression of BMPR2 in placentas from mothers with hypertension (see Table 2). Previous data show that BMPR2 and these ligands are critical for the maintenance of vascular development during pregnancy via VEGF production and invasion of the uterine wall and embryo placentation (Nagashima et al., 2013; You et al., 2021). Previous data suggest that Heparanase and BMPR2 can play a potential role during maternal hypertension in the placenta via inhibiting the proangiogenic effects of VEGF.

Another gene associated with the VEGF receptor signaling pathway (Table 2) is Rho-associated coiled-coil-containing protein kinase-2 or ROCK2. It is a serine/threonine kinase expressed at a high level in the placenta from preeclamptic women, inducing actin cytoskeleton rearrangement in the trophoblast cell and shedding of Syncytiotrophoblast macrovesicles and exosomes, accompanied with a decreased outgrowing microvilli (Han et al., 2016). Similarly, heat-shock 27-KD protein-1 or HSPB1 plays a role in the VEGF receptor signaling pathway and shows an impaired expression in pathological placenta with hypertension (Table 2). Low expression of Het shock protein HSPB1 and HSP70 play a role in vascular alteration, and the umbilical artery flow modification detected in the placenta after premature birth (Dvorakova et al., 2017), which suggests that the relevance of different modulators can change the VEGF pathway in pathological placentas.

G protein-coupled estrogen receptor-1 (GPER1) proteins modify their expression in the placenta of mothers with hypertension (Table 2). They are a mediator of estrogen signaling and protect the fetus during maternal inflammation and are associated with negative regulation of vascular smooth muscle cell proliferation. For example, GPER1 protein can prevent the adverse effects of type-I Interferon during maternal infection (Harding et al., 2021). Moreover, the level of GPER1 in placentas from preeclampsia reduced by about 50%, a reduction that can partially be associated with estrogen treatment in trophoblast culture (Feng et al., 2017), which correlates with elevated apoptosis and minor cellular proliferation in the placenta from preeclampsia (Li et al., 2016). The functions of some of the genes detected in Table 2 and their relation with the vascular disease require further study to better understand the role of these genes during hypertensive pathology during pregnancy and their role in the placenta.

Several reports suggest that melatonin plays a role as a protector agent during pregnancy for the mother, fetus, and placenta physiology (Dou et al., 2019; Nagasawa et al., 2021; Sun et al., 2021). Melatonin could directly stabilize blood pressure in pathological pregnancies via modifying previously described genes or new targets, which requires more studies to be conducted during gestational hypertension, preeclampsia, and other pathologies.

## Melatonin and Hypertension

Melatonin receptors are associated with the activation of two G-protein-coupled receptors named MT1 and MT2, which, via Gi-and Gq-receptor activation, lead to decreased levels of cAMP and increased levels of cytosolic calcium. Both receptors participate in the temporal synchronization of the circadian system and sleep quality (Jockers et al., 2016). Diurnal animals and humans have shown high blood pressure during daytime hours, and a dip of about 10-20% during darks hours such as human correlated melatonin secretion. Similarly, circadian rhythms have been observed for heart rate, which is abolished by the impaired secretion of the pineal hormone (Fabbian et al., 2013; Tabara et al., 2018). In contrast, the absence of circadian rhythms of blood pressure elevates the risk for cardiovascular morbidity/mortality by ventricular hypertrophy, renal dysfunction, remodeling of carotid structure, cerebrovascular accident, hypertension, and stroke (Baker and Kimpinski, 2018). The relevance of these circadian rhythms can be observed in the pharmacological treatment of hypertension with an angiotensin II receptor blocker, which is more effective during the higher production of melatonin hormone or night hours (Giles, 2006), suggesting the relevance of circadian rhythms and melatonin signaling for cardiovascular health.

A correlation between high blood pressure and arterial stiffness has been observed in patients, and the risk is higher when the disruption of the circadian rhythms of blood pressure is more severe. This severity is associated with the minor amplitude of the circadian rhythms or, eventually, a flattening of the circadian pattern, not showing a lower systolic or diastolic pressure during the night hours (Park et al., 2019). For example, after liver transplantation, about 90% of patients observed a chronodisruption of blood pressure oscillation, with about 55% showing an arrhythmic pattern and about 36% showed an inverted pattern. This chronodisruption has been associated with poor glomerular filtration of Cystatin-C and plasmatic accumulation (Hryniewiecka et al., 2018), the latter being a marker for robust kidney injury, systemic inflammation, and mortality (Hendrickson et al., 2020).

The melatonin receptor is present in several vascular tissues such as the Circle of Willis and vertebral arteries, the caudal artery, aorta, coronary arteries and carotids, cardiac ventricular wall, and systemic arteries, suggesting that melatonin plays a role in various cardiovascular diseases (Baker and Kimpinski, 2018; Prado et al., 2018). For example, a low level of melatonin is detected in Coronary heart disease (5-fold), elevating the risk of infarction and death. This can occur because the suppression of melatonin production induces vascular vasoconstriction and hypertension, and their supplementation reduces the blood pressure, inflammation, vascular infiltration of lymphocytes, aldosterone levels, and lowers the risk of deaths caused by myocardial infarction via reduction of oxidative stress (Baker and Kimpinski, 2018; Prado et al., 2018; Simko et al., 2018). Similarly, newborn sheep supplemented with melatonin showed reduced pulmonary arterial pressure. Moreover, the elevation of the vascular vasodilatation, which occurs via elevation of antioxidant capacity by stimulation of antioxidant activity SOD, CAT, GPx, causes induction of vasodilator genes and inhibition of vasoconstrictor gene response (Gonzaléz-Candia et al., 2020), suggesting the ubiquitous effects of this hormone in several vascular territories, lowering the risk of cardiovascular disease.

Interestingly, patients with pulmonary hypertension showed a low plasmatic level of melatonin and elevated levels of IL-1β. When analyzing animal models, supplementation with melatonin inhibits hypoxia-induced thickness and the remodeling of the pulmonary artery. It reduced the expression level of cytokine proinflammatory IL-1β in pulmonary tissue 3-fold and reduces macrophage activation (Zhang et al., 2020). A similar result was observed in gestational hypertension induced by L-NAME, melatonin supplementation can lower systolic blood pressure by about 10% and urine protein content by about 30%, increasing the antioxidant capacity of rats by about 28%, and lowering the sFlt-1 level circulation in about 29% of cases (Zuo and Jiang, 2020).

A study in patients with type 2 diabetes and hypertension demonstrated that about 30-32% of non-dipping people treated with 3-5 mg of melatonin saw a restoration of the dipping for systolic blood pressure, diastolic blood pressure, and mean arterial pressure during the dark hours, suggesting that melatonin could synchronize the circadian oscillation of blood pressure in about one-third of patients (Możdżan et al., 2014). A similar observation was seen in animal models where melatonin administration reduced hypertension in animals with metabolic syndrome (Baker and Kimpinski, 2018). Moreover, melatonin has the dual capacity to modulate vascularization depending on the cellular condition. For example, in pathological tissues exposed to a lesion, melatonin induces angiogenesis, such as skin, bone, and gastric ulcers. This effect occurs because melatonin induces endothelial expression and secretion of VEGF, stimulating neovascularization (Ma et al., 2020).

Patients with dyslipidemia and atherosclerosis showed a low level of melatonin production, which lowers the plasmatic level of fibrinogen, FVIII, and leads to the inhibition of platelet aggregation (Otamas et al., 2020). A similar observation was made in postmenopausal women with prevalent hypertension, which showed a reduction of 26% in the urinary metabolite of melatonin 6-Sulfatoxy-Melatonin, and this chronic low-level melatonin elevates the risk of hypertension by about 17-23%. The risk was elevated by 60% when the patient reported using alcohol or medication to sleep (Pérez-Caraballo et al., 2018). A critical element for inducing vascular vasodilation is nitric oxide, which can be induced by melatonin, producing vasodilation, lowering blood pressure, and reducing Endothelin and Angiotensin II effects on humans umbilical vein endothelial cells (Baker and Kimpinski, 2018).

Hypertension and valvular dysfunction can induce heart failure and hypertrophy. The aortic constriction induces cardiac hypertrophy markers of natriuretic protein ANP, BNP, and β-MHC. However, these can be reverted by melatonin supplementation. Similarly, the apoptosis markers, caspase-3, cytochrome-c, and Bax, and the autophagy are lowered by melatonin treatment, suggesting the protective role of melatonin after aortic constriction. This inhibition of cardiac hypertrophy can occur due to the capacity of melatonin to stimulate the protein activation of p-mTOR, p-AKT, and the activation of the down pathways p-S6K and p-4E-BP1(Xu et al., 2020).

Several unknown pathways could potentially explain some of the effects of melatonin. For the functional analysis of the pathways correlated between melatonin and hypertension, we performed the search Analysis of Datasets of the GEO database^2^. Queries were performed using the “MELATONIN” keyword after a systematic SEARCH restricted to specific fields. We downloaded 39 experimental results for melatonin, and excluded the platforms without gene accession numbers, incomplete incoming information, cancer cells, transgenic animals, knockout, or experiments with modification of photoperiod. We then obtained experimental platforms “GSE92612” and “GSE169459” with vascular smooth muscle contraction pathways modified by melatonin. We observed 115 common genes, see Table 3. Furthermore, the genes that were combined and identified with Venn Diagram showed 35 common elements between “Hypertension” (Table 1) and “Melatonin,” see Table 4. The differentially expressed genes obtained here showed several examples modified by melatonin over the vascular tone, which require further study into their potential role in hypertensive pathology during the pregnancy and their therapeutic role in protecting the placenta/fetus/mother from the adverse effects of hypertension.

**TABLE 3:** Vascular smooth muscle contraction pathways modified by melatonin supplementation.

Differentially expressed genes associated with melatonin (*N* = 115 genes)					
ARAF	ADCY3	CALM2	MYLK2	PLA2G10	PRKACA
BRAF	ADCY4	CALM3	MYLK3	PLA2G12A	PRKACB
GNA11	ADCY5	CALML3	MYLK4	PLA2G12B	PRKACG
GNA12	ADCY6	CALML5	MYLK	PLCB1	PRKG1
GNA13	ADCY7	CALML6	NPR1	PLCB3	PPP1CA
GNAQ	ADCY8	EDNRA	NPR2	PLCB4	PPP1CB
GNAS	ADCY9	GUCY1A2	PLA2G1B	KCNMA1	PPP1CC
JMJD7-PLA2G4B	ADRA1A	GUCY1A3	PLA2G2A	KCNMB1	PPP1R14A
RAF1	ADRA1B	GUCY1B3	PLA2G2C	KCNMB2	PPP1R12A
ROCK1	ADRA1D	ITPR1	PLA2G2D	KCNMB3	PPP1R12B
ROCK2	AGTR1	ITPR2	PLA2G2E	KCNMB4	PPP1R12C
ARHGEF1	AVPR1A	ITPR3	PLA2G2F	KCNU1	RHOA
ARHGEF11	AVPR1B	MAPK1	PLA2G3	PTGIR	RAMP1
ARHGEF12	CALCRL	MAPK3	PLA2G4A	PRKCA	RAMP2
ACTA2	CACNA1C	MAP2K1	PLA2G4C	PRKCB	RAMP3
ACTG2	CACNA1D	MAP2K2	PLA2G4D	PRKCD	
ADORA2A	CACNA1F	MRVI1	PLA2G4E	PRKCE	
ADORA2B	CACNA1S	MYL6	PLA2G4F	PRKCH	
ADCY1	CALD1	MYL6B	PLA2G5	PRKCG	
ADCY2	CALM1	MYL9	PLA2G6	PRKCQ	

*Functional analysis of pathways of melatonin by analysis of datasets from the GEO database (http://www.ncbi.nlm.Nih.gov/geo). The systematic search was restricted to the following specific fields: expression profiling by the array. In total, 39 experimental results for melatonin were downloaded, and we excluded the platforms without gene accession number, incomplete incoming information, cancer cells, transgenic animals, knockout, or experiments with modification of photoperiod. After this revision, we obtained experimental platforms “GSE92612” and “GSE169459,” which were analyzed by GEO2R and selected vascular smooth muscle contraction pathways. Both experiments had 115 common genes.*

**TABLE 4:** Correlation of vascular smooth muscle contraction pathways modified by hypertension and melatonin supplementation.

Differentially expressed genes associated with hypertension *and melatonin (N* = *35 genes)*			
*ARAF*	*GUCY1B3*	*PLA2G2A*	*PPP1CC*
*GNAS*	*ITPR1*	*PLA2G4A*	*RHOA*
*RAF1*	*ITPR2*	*PLA2G6*	*RAMP1*
*ARHGEF11*	*ITPR3*	*PLA2G2C*	*RAMP2*
*ADCY2*	*MAPK1*	*PLA2G5*	*RAMP3*
*ADCY5*	*MAP2K1*	*PLCB4*	
*ADCY6*	*MAP2K2*	*KCNMB4*	
*ADRA1D*	*MYLK2*	*PRKCD*	
*CALM3*	*NPR1*	*PRKCH*	
*GUCY1A3*	*NPR2*	*PRKCG*	

*Analysis by Venn Diagram from Tables 1, 3. We identified 35 common elements between “Hypertension” and “Melatonin” associated with vascular smooth muscle contraction.*

### Melatonin and Pregnancy

Several studies reported a relationship between hypertension and melatonin, inducing negative outputs during pregnancy via modifying the endothelial function, antiplatelet effects, vascular tone, vasoactive factor production, and oxidative stress. For example, during severe preeclampsia, melatonin production and the expression of MT1 and MT2 receptors are lower than in normal pregnancies. Moreover, the supplementation of melatonin to patients can delay the delivery and reduce oxidative stress and hypertension during pregnancy (de Chuffa et al., 2019). VEGF production during normal pregnancy is inhibited when the mother produces a low level of melatonin, increasing the risk of premature birth and abortion (Valenzuela et al., 2015; de Chuffa et al., 2019). During normal pregnancy, a progressive increase in melatonin levels is observed, and non-dipper blood pressure pregnant women with preeclampsia showed more severe hypertension correlated to a minor level of melatonin production during dark hours (Bouchlariotou et al., 2014). The placenta is an extra-pineal gland site for the synthesis of melatonin hormones, and placentas from preeclampsia have shown a low level of expression of the critical enzyme of melatonin synthesis in placenta AA-NAT and HIOMT. Interestingly, melatonin supplementation has antioxidant effects on the placenta and reduces the levels of sFlt1, Activin-A, and sEng, reducing trophoblastic debris from the early trimester placentae exposed to preeclamptic serum. The trophoblast mitochondria synthesize melatonin locally, protecting the mitochondrial and the respiratory function, a critical protagonist during placental hypoxia induced by preeclampsia (Langston-Cox et al., 2021). It prevents the oxidative stress of the placenta, inducing the activation of the antioxidant system via elevation of Nrf-2 translocation, a potent inductor of mitochondrial activity and biogenesis (Hobson et al., 2018). Melatonin supplementation during pregnancy in animal models increases umbilical blood (Thakor et al., 2010; Langston-Cox et al., 2021), protecting the endothelial function, repairing the endothelial monolayer, inhibiting vascular inflammation and VCAM-1 production in placentas obtained from preeclamptic women, and reducing blood pressure and sFLT-1, markers of vascular damage during preeclampsia (Hung et al., 2013; Reiter et al., 2017; Hobson et al., 2018; de Chuffa et al., 2019). Additionally, in women with early onset of preeclampsia, melatonin supplementation prolonged the interval from diagnosis to delivery in 6 days and required minor doses of antihypertensive treatment (Hobson et al., 2018), suggesting the partial inhibition of adverse effects of preeclampsia.

**FIGURE 2:**
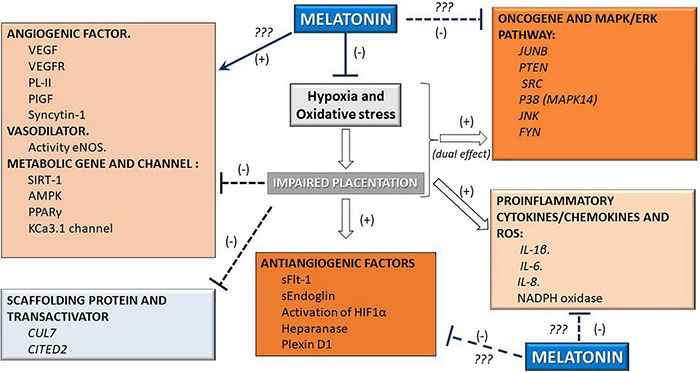
Melatonin can modify molecular pathways in the placenta. Given the supraphysiological effect of the melatonin hormone, several pathways that affect vascular function are inhibited (-) or stimulated (+) in the placenta. These effects involve the hypoxia, pro/antiangiogenic, vasodilatory, metabolic, oncogenic, antioxidant, and proinflammatory pathways.

The umbilical blood sample collected at term from pregnancies affected by intrauterine growth restriction, or IUGRA, showed a lower level of melatonin circulation (∼50%). This reduction occurs parallel to the reduced circulatory levels of the angiogenic factor PIGF observed in the umbilical blood (Hobson et al., 2018; Berbets et al., 2020). The supplementation of melatonin in an animal model of gestational hypertension can lower the systolic blood pressure and urine protein content and ameliorate placental weight reduction. Moreover, it reduces the antiangiogenic production of sFLT-1, increases the proangiogenic factor PIGF, and increases the mother’s antioxidant capacity (Zuo and Jiang, 2020). Similarly, melatonin reverted partially placental impaired perfusion, placental coagulation, and induced anti-inflammatory factors in mouse pregnancy associated with intrauterine inflammation-related oxidative stress (Lee et al., 2019). Reduction of oxidative stress and improvement of the placental perfusion induced by melatonin can occur by an improved endothelial function via increased nucleus translocation of Nrf2 and elevation of endogenous antioxidant enzymes heme-oxygenase-1 (Hobson et al., 2018). These antecedents suggest the hormone melatonin’s various actions on placental function and its potential role in modulating several modified pathways in pathological pregnancies (see Figure 2).

## Conclusion

Melatonin hormone has antioxidant, homeostatic, and time-giving roles at the level of the vascular system. The temporal desynchronization of the vascular system by inhibition of melatonin production induces pathology such as hypertension. Melatonin supplementation shows a protective role over the vascular system, reverting elevation of blood pressure, oxidative stress, and antiangiogenic factors. During pregnancy, impaired production of melatonin can elevate the risk of poor fetal/placental development by preeclampsia, intrauterine growth restriction, and preterm birth. Melatonin can protect the pregnancy via stimulation of the antioxidant system, vascular factors such as VEGF, PIGF, and by inhibiting antiangiogenic factors such as sFLT-1 and sEng. Current evidence describes an elevation of melatonin production during pregnancy by the placenta, and we believe that local production is a keystone molecule in placental physiology. In this regard, we propose that melatonin plays a supraphysiological and dual role over placental physiology and could be the future for the protection and therapeutic application of vascular pathologies of pregnancies.

## Author Contributions

FV-M, CL, and GD contributed to conception and analysis. KJ-M and CL organized the database. FC-P and FV-M performed the bioinformatic analysis. FV-M wrote the first draft of the manuscript. All authors contributed to manuscript revision, read, and approved the submitted version.

## Conflict of Interest

The authors declare that the research was conducted in the absence of any commercial or financial relationships that could be construed as a potential conflict of interest.

## Publisher’s Note

All claims expressed in this article are solely those of the authors and do not necessarily represent those of their affiliated organizations, or those of the publisher, the editors and the reviewers. Any product that may be evaluated in this article, or claim that may be made by its manufacturer, is not guaranteed or endorsed by the publisher.

## Footnotes

1
https://www.ncbi.nlm.nih.gov/gds

2
http://www.ncbi.nlm.Nih.gov/geo
